# 

**Published:** 1913-10

**Authors:** 


### CONTENTS.

ORIGINAL COMMUNICATIONS—

The Early Treatment of Certain Diseases, by A. W. Stirling, M. D., C. M. (Edin.), D. P. H. (Eng.), Opthalmic Surgeon to Grady Hospital, etc. Atlanta, Ga. ---------- 287
The Diagnostic Significance of Convulsions, by Lewis M. Gaines, M. D., Prof. Neurology and Mental Diseases, Atlanta Medical College, Atlanta, Ga. 293
Non-Surgical Treatment of the Accessory Sinuses, by Richard R. Daly, M. D., Atlanta, Ga. ---------- 298
Some Diseases of the Female Urethra, by Dr. W. F. Shallenberger, Atlanta, Ga. ---------- 303
Uses of Physiologic Therapy. Theodore Toepel, M. D., Atlanta, Ga. ---------- 308
Hydrotherapy in the Home. J. H. Neal, M. D., Atlanta, Ga., ---------- 310
Repair of the Perineum, Using Silk-Worm Gut aud Leaving the Sutures In for Two or Three Months, by Gaston Torrance, M. D., Birmingham, Ala.. 315
EDITORIAL ---------- 316
SELECTIONS AND ABSTRACTS ---------- 318

